# A tutorial on information retrieval: basic terms and concepts

**DOI:** 10.1186/1747-5333-1-2

**Published:** 2006-03-13

**Authors:** Wei Zhou, Neil R Smalheiser, Clement Yu

**Affiliations:** 1 Department of Computer Science, University of Illinois at Chicago, 851 South Morgan Street, Chicago, IL 60607, USA; 2 Department of Psychiatry and Psychiatric Institute, MC912, University of Illinois at Chicago, Chicago, IL 60612, USA

## Abstract

This informal tutorial is intended for investigators and students who would like to understand the workings of information retrieval systems, including the most frequently used search engines: PubMed and Google. Having a basic knowledge of the terms and concepts of information retrieval should improve the efficiency and productivity of searches. As well, this knowledge is needed in order to follow current research efforts in biomedical information retrieval and text mining that are developing new systems not only for finding documents on a given topic, but extracting and integrating knowledge across documents.

## Introduction

Biomedical researchers use PubMed and Google everyday, and the success of these search engines is closely linked to the fact that one does not need to know how the systems work in order to obtain useful answers to queries that are posed. Nevertheless, scientists tend to be mechanistic thinkers, so they may naturally become curious about the mechanisms that underlie these services. Also, even if a neophyte can get acceptable results, knowing the information retrieval technology underlying these search engines should allow one to get better results more efficiently [1,2]. Finally, if indeed "biology has become an information science" [3], then all investigators should have a working knowledge of basic information retrieval (IR) terms and concepts, even those who are not engaged in IR research themselves.

### PubMed

PubMed [4] is a service of the National Library of Medicine that includes over 15 million bibliographic citations from MEDLINE and other life science journals for biomedical articles back to the 1950s. The full text of articles are not stored; rather, links to the provider's site to obtain the full-text of articles are given, if available. Each article is indexed according to multiple fields, including title, abstract, author names, journal name, language of publication, year of publication, etc. (Table 1). Each article in MEDLINE is also indexed using a controlled vocabulary, called **Medical Subject Headings **(MeSH), which is used to describe the main topics discussed [5]. The set of MeSH terms is manually assigned by biomedical experts who scan each article.

**Table 1:** A citation record that shows the MEDLINE fields. Fukagawa T, Nogami M, Yoshikawa M, Ikeno M, Okazaki T, Takami Y, Nakayama T, Oshimura M. Dicer is essential for formation of the heterochromatin structure in vertebrate cells. Nat Cell Biol. 2004 Aug;6(8):784-91.

PMID	15247924
OWN	NLM
STAT	MEDLINE
DA	20040810
DCOM	20040827
LR	20051122
PUBM	Print-Electronic
IS	1465–7392 (Print)
VI	6
IP	8
DP	2004 Aug
TI	Dicer is essential for formation of the heterochromatin structure in vertebrate cells.
PG	784-91
AB	RNA interference is an evolutionarily conserved gene-silencing pathway in which the nuclease Dicer cleaves double-stranded RNA into small interfering RNAs. The biological function of the RNAi-related pathway in vertebrate cells is not fully understood. Here, we report the generation of a conditional loss-of-function Dicer mutant in a chicken-human hybrid DT40 cell line that contains human chromosome 21. We show that loss of Dicer results in cell death with the accumulation of abnormal mitotic cells that show premature sister chromatid separation. Aberrant accumulation of transcripts from alpha-satellite sequences, which consist of human centromeric repeat DNAs, was detected in Dicer-deficient cells. Immunocytochemical analysis revealed abnormalities in the localization of two heterochromatin proteins, Rad21 cohesin protein and BubR1 checkpoint protein, but the localization of core kinetochore proteins such as centromere protein (CENP)-A and -C was normal. We conclude that Dicer-related RNA interference machinery is involved in the formation of the heterochromatin structure in higher vertebrate cells.
AD	Precursory Research for Embryonic Science and Technology of Japan Science and Technology Agency, National Institute of Genetics and The Graduate University for Advanced Studies, Mishima, Shizuoka 411-8540, Japan. tfukagaw@lab.nig.ac.jp
FAU	Fukagawa, Tatsuo
AU	Fukagawa T
FAU	Nogami, Masahiro
AU	Nogami M
FAU	Yoshikawa, Mitsuko
AU	Yoshikawa M
FAU	Ikeno, Masashi
AU	Ikeno M
FAU	Okazaki, Tuneko
AU	Okazaki T
FAU	Takami, Yasunari
AU	Takami Y
FAU	Nakayama, Tatsuo
AU	Nakayama T
FAU	Oshimura, Mitsuo
AU	Oshimura M
LA	eng
PT	Journal Article
DEP	20040711
PL	England
TA	Nat Cell Biol
JT	Nature cell biology.
JID	100890575
RN	0 (Cell Cycle Proteins)
RN	0 (Heterochromatin)
RN	0 (Nuclear Proteins)
RN	0 (Phosphoproteins)
RN	0 (RAD21 protein, human)
RN	EC 2.7.1.- (Bub1 spindle checkpoint protein)
RN	EC 2.7.1.37 (Protein Kinases)
RN	EC 3.1.- (Endoribonucleases)
SB	IM
CIN	Nat Cell Biol. 2004 Aug;6(8):696-7. PMID: 15303098
MH	Animals
MH	Blotting, Western
MH	Cell Cycle Proteins/genetics/metabolism
MH	Cell Death/genetics
MH	Cell Line
MH	Cell Survival
MH	Centromere/chemistry
MH	Chickens
MH	Chromosomes, Human, Pair 21
MH	Endoribonucleases/deficiency/*genetics/*physiology
MH	Gene Silencing
MH	Heterochromatin/*chemistry/genetics/*metabolism
MH	Humans
MH	Immunohistochemistry
MH	In Situ Hybridization, Fluorescence
MH	Models, Biological
MH	Mutation
MH	Nuclear Proteins/genetics/metabolism
MH	Phosphoproteins/genetics/metabolism
MH	Protein Kinases/genetics/metabolism
MH	RNA Interference
MH	Research Support, Non-U.S. Gov't
MH	Restriction Mapping
MH	Transgenes
EDAT	7/13/2004 5:00
MHDA	8/28/2004 5:00
PHST	2004/05/29 [received]
PHST	2004/06/29 [accepted]
PHST	2004/07/11 [aheadofprint]
AID	10.1038/ncb1155 [doi]
AID	ncb1155 [pii]
PST	ppublish
SO	Nat Cell Biol. 2004 Aug;6(8):784-91. Epub 2004 Jul 11.

**Figure 1:**
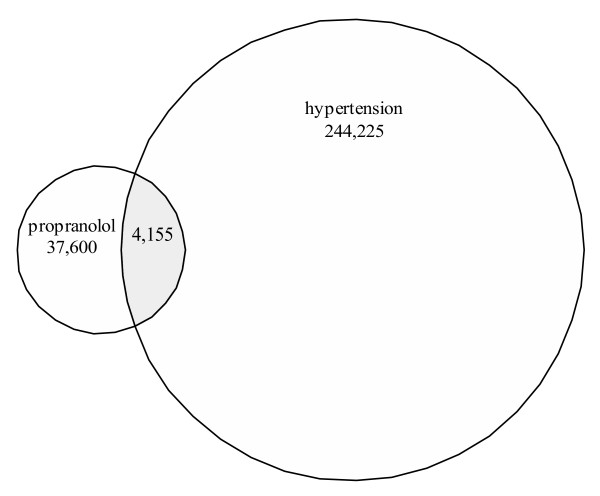
**Venn diagram visualization of a PubMed search**. 37,600 documents were retrieved when "propranolol" was searched in PubMed and 244,225 for "hypertension". The overlap, 4,155 documents, is the set of documents having both "propranolol" and "hypertension".

Let us consider a particular information need: A user would like to identify recent articles that discuss the use of propanolol in hypertension. First, he/she must translate this information need into a **query **in the correct format so that it can be processed correctly. PubMed employs the **Boolean operators **AND, OR, and NOT. The AND operator is used to retrieve a set in which each record contains all the search terms. This operator places no condition on where the terms are found in relation to one another; the terms simply have to appear somewhere in the same record. For example, if one desired documents on the use of the drug propanolol in the disease hypertension, a typical search statement might be [propanolol AND hypertension] (the brackets are used to delimit the query but are not part of the query itself) (Fig. 1). The OR operator retrieves documents that contain at least one of the specified search terms. The NOT operator excludes the specified terms from the search. Certain very common words (e.g., "this") are placed on a **stoplist **and are automatically excluded from queries.

Before PubMed begins to retrieve articles, it performs **query preprocessing**, to identify which fields of the MEDLINE record are relevant, and to alter or expand the query terms via **automatic term mapping**. For example, the query [high blood pressure] will be automatically mapped to the MeSH term "hypertension" (each MeSH term may have a set of synonyms as alternative entry terms. In this example, "high blood pressure" is one of the synonyms or entry terms of "hypertension"). PubMed will search using the mapped MeSH term within the MeSH field, as well as the term originally entered. MeSH comprises a hierarchy of terms, and the more specific terms corresponding to that MeSH term will also automatically be searched. In the above example, three more specific MeSH terms "Hypertension, Malignant", "Hypertension, Renal", and "Hypertension, Pregnancy-Induced" are also searched. This process of adding related terms is called **query expansion**. It is basically an OR operation. PubMed also maintains a **phrase list**, so that commonly used phrases are recognized and handled as such. After query preprocessing, PubMed looks for **exact matches **between the query terms and terms within the specified MEDLINE fields, and returns a list of documents ranked in reverse chronological order (optionally, by first author's last name or by journal name). A more detailed PubMed tutorial can be found at [6], and tips for MEDLINE searching can be found at [7].

### Google

Unlike PubMed, which is a search engine limited to biomedical literature, Google searches billions of web pages. However, like PubMed, Google uses a Boolean search strategy. Submitting a query to Google in the form: [propanolol hypertension] will return all the web pages that match both "propanolol" and "hypertension" exactly (though some of the terms may reside not on the retrieved page itself, but on pages that link to the retrieved page). Google also supports OR and NOT Boolean operators. Instead of returning web pages chronologically, Google employs a unique method called PageRank and sophisticated text-matching techniques to find pages that are both important and relevant to a query.

**PageRank **is a method for measuring the importance of web pages developed by Larry Page and Sergey Brin [8]. PageRank relies on the link structure of the web as an indicator of an individual page's value. If many web pages have links to page A, then page A is given more weight. But, Google looks at more than the sheer volume of links a page receives; it also analyzes the page that contains the link. Links by pages that are themselves "important" weigh more heavily and help to make other pages "important" (see Table 3 for details). Google also uses text-matching techniques to measure the similarity between a query and web pages [9]. Google considers over a hundred factors in determining which web pages are most relevant to a query; for example, it gives higher ranks to pages that have search terms near each other and in the same order as the query, or pages that have search terms in important sections of a web page (such as title).

**Table 2:** Term weighting and normalization in the vector space model.

A typical term weighting strategy combines the **inverse document frequency **(IDF) and **term frequency **(TF). They are defined as:
TF(term, document) = frequency of term in document WEIGHT(term, document) = TF(term, document) * IDF(term)
The idea of IDF is that the fewer documents having the term, the more useful the term is in discriminating those documents having it from those not having it. On the other hand, if a term occurs many times in a document, then it is likely that the term is significant in representing the contents of the document. With this weighting strategy, the highest weight is accorded to terms that occur frequently in a document but infrequently elsewhere.
With very large collections, not all terms in the document are used for indexing. Some terms have to be removed. This is usually accomplished through the elimination of **stopwords **(such as articles and connectives), or the use of **stemming **(which reduces distinct words to their common grammatical root). **Porter stemming **[27] is probably the most widely used stemming algorithm in the IR community.
The most common approach to relevance ranking in VSM is to give each document a score based on the sum of the weights of terms common to the document and query. Terms in documents typically derive their weight from the TF*IDF. Then the similarity between each document and the query is computed with the formula:

One problem with TF*IDF weighting is that longer documents accumulate more weight in queries simply because they have more words. As such, some approaches "normalize" the weight of a document. The most common approach is **cosine normalization **[28]:

A variety of other variations to the basic VSM have been developed. For example, **Okapi weighting **is based on the Poisson distribution [29]. Another variation of TF*IDF document weighting, **pivoted normalization**, is also often used [30].

**Table 3:** The Google PageRank algorithm.

PageRank is defined as follows [8]:
We assume web page A has pages T_1_...T_n _which link to it. The parameter d is a damping factor which can be set between 0 and 1 (usually set to 0.85). Also, C(A) is defined as the number of links going out of page A. The PageRank of a page A is given as follows:
PR(A) = (1-d) + d (PR(T_1_)/C(T_1_) + ... + PR(T_n_)/C(T_n_))
PageRanks form a probability distribution over web pages. The PageRank value of a web page reflects the frequency of encountering that page by a Web user who surfs across the web following links randomly.

### Vector space model

Users cannot simply enter Boolean queries the way they would normally write or speak (**free text**), because the Boolean logic incorporates all terms used – thus, extra or colloquial words might unduly restrict or expand the query. Submitting a query to PubMed in the form: [tell me what are the indications for propranolol in hypertension] retrieves no articles, because authors are not likely to use "tell" or "me" in their academic articles. (NLM does maintain an experimental MEDLINE interface for free text queries at [10].) **Partial-matching **will allow free text queries and retrieve all the documents that have at least one of the search terms and then rank them according to their relevance to the query.

The classic IR model of partial-matching is the **vector space model **(VSM), which is usually attributed to Salton [11]. In the VSM, each document is represented, or **indexed**, by a vector of weighted terms. For example, the query [tell me what are the indications for propranolol in hypertension] may be represented by a vector including the terms "indications", "propranolol", and "hypertension". (The words "tell", "me", "for", and so on may be eliminated if a stoplist is employed.) Each document in the collection to be searched (e.g. web pages indexed by Google) is represented in the same manner. Often **stemming **is employed to recognize variants of the same word, which will also reduce the number of terms indexed. For example, the words "beautiful" and "beautify" come from the same stem, "beauty". Thus they are often identified by one term only. Notice that the VSM does not capture all features of a document or a query. For example, the ordering of the terms in a document is not recorded, so "a cat chases a mouse" will be indistinguishable from "a mouse chases a cat". The terms are usually **weighted **in terms of their importance. A common weighting strategy is to assign high weights to terms that occur frequently in a document but infrequently elsewhere.

The **similarity **between each document stored in the system and the user query is defined as the difference between the document vector and the query vector. Documents are typically ranked by their closeness of fit to the query. This is called **relevance ranking **(see Table 2 for details).

### Refining searches

Although searches are easy to carry out, it is notoriously difficult to craft a query that exactly captures the user's intent on the first try. **Relevance feedback **is a technique commonly used to improve retrieval performance [12,13]. It is a process in which the user conducts an initial query, and then provides feedback as to what documents are relevant. Terms from those known relevant document are then added to the query. Alternatively, the search engine can attempt to identify relevant documents automatically by assuming that the top-ranked documents are relevant, so that new terms are selected automatically (this is known as **pseudo relevance feedback**, or **blind query expansion**). A new query is formulated by adding the selected terms for a second round retrieval. Through a query expansion, some relevant documents missed in the initial round can then be retrieved to improve the overall performance.

### Evaluating the performance of search engines

An IR system returns a ranked list of documents to a user's query. How does the result satisfy the user? The most widely used measure is the relevance-based measure of **recall **and **precision**. With respect to a given query, the entire space of documents can be partitioned into four sets: Relevant to the user and retrieved by the system; relevant but not retrieved; irrelevant and retrieved; irrelevant and not retrieved. The recall and precision are defined based on these four sets.





Recall indicates what proportion of all the relevant documents have been retrieved from the collection. Precision indicates what proportion of the retrieved documents is relevant. One of the problems with this measure is that the total number of relevant documents in the collection is usually unknown. Thus, a **gold standard**, where all the documents are judged as relevant or irrelevant to each query, is usually constructed manually by evaluators to use this measure. In a reasonably sized collection, it is impossible to judge all documents with respect to each query. Instead, "pooled relevance assessment" is carried out in which multiple search engines are used, each one retrieving a certain number of documents for each query. The assessors judge the union of the retrieved documents, or sometimes, the commonly retrieved documents.

**Figure 2:**
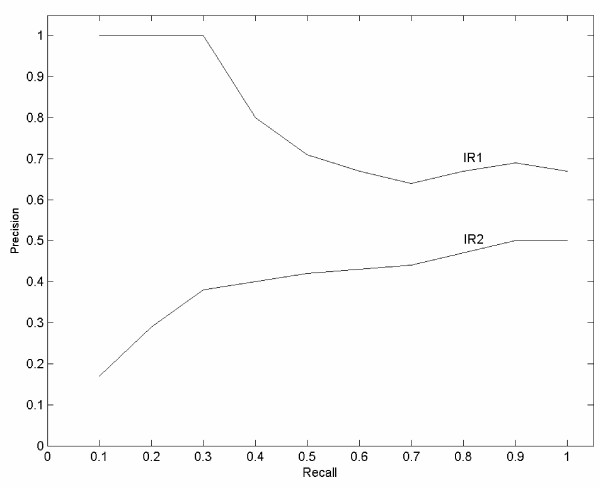
**Curve of precision vs. recall for the two IR systems shown in Table 4**. IR systems typically show a trade-off between recall and precision, so that the more documents that are retrieved, the more irrelevant documents will be included. On the other hand, it can be seen that system IR1 performs uniformly better than system IR2 since it has higher precision values at every recall level.



In order to combine recall and precision into a single overall measure, some workers employ a recall-precision table, where 11 intervals from a recall of 0.0 to 1.0 are used and the average of precision at each point of recall is reported as the summary result. This is also called **11-point average precision **(Table 4, Fig. 2). Another approach to combine recall and precision is the ***F*measure**. A simple version of F measure is as follows:

**Table 4:** 11-point precision diagram. This example shows a query that is submitted to two different IR systems (IR1 and IR2), which are based on the same collection of 20 documents. Both IR1 and IR2 rank all 20 documents, of which 10 are relevant. However, IR1 ranks the relevant documents higher on average than does IR2. The mean average precision for IR1 = 0.79 and for IR2 = 0.40. The recall and precision curves for IR1 and IR2 are shown in figure 2.

Ranking by IR1					Ranking by IR2				
Ranking	Doc	Relevant	Recall	Precision	Ranking	Doc	Relevant	Recall	Precision

**1**	**d**_**1**_	**yes**	**0.10**	**1.00**	1	d_1_	no	0.00	0.00
**2**	**d**_**2**_	**yes**	**0.20**	**1.00**	2	d_2_	no	0.00	0.00
**3**	**d**_**3**_	**yes**	**0.30**	**1.00**	3	d_3_	no	0.00	0.00
4	d_4_	no	0.30	0.75	4	d_4_	no	0.00	0.00
**5**	**d**_**5**_	**yes**	**0.40**	**0.80**	5	d_5_	no	0.00	0.00
6	d_6_	no	0.40	0.67	**6**	**d**_**6**_	**yes**	**0.10**	**0.17**
**7**	**d**_**7**_	**yes**	**0.50**	**0.71**	**7**	**d**_**7**_	**yes**	**0.20**	**0.29**
8	d_8_	no	0.50	0.63	**8**	**d**_**8**_	**yes**	**0.30**	**0.38**
**9**	**d**_**9**_	**yes**	**0.60**	**0.67**	9	d_9_	no	0.30	0.33
10	d_10_	no	.060	0.60	**10**	**d**_**10**_	**yes**	**0.40**	**0.40**
**11**	**d**_**11**_	**yes**	**0.70**	**0.64**	11	d_11_	no	0.40	0.36
**12**	**d**_**12**_	**yes**	**0.80**	**0.67**	**12**	**d**_**12**_	**yes**	**0.50**	**0.42**
**13**	**d**_**13**_	**yes**	**0.90**	**0.69**	13	d_13_	no	0.50	0.38
14	d_14_	no	0.90	0.64	**14**	**d**_**14**_	**yes**	**0.60**	**0.43**
**15**	**d**_**15**_	**yes**	**1.00**	**0.67**	15	d_15_	no	0.60	0.40
16	d_16_	no	1.00	0.63	**16**	**d**_**16**_	**yes**	**0.70**	**0.44**
17	d_17_	no	1.00	0.59	**17**	**d**_**17**_	**yes**	**0.80**	**0.47**
18	d_18_	no	1.00	0.56	**18**	**d**_**18**_	**yes**	**0.90**	**0.50**
19	d_19_	no	1.00	0.52	19	d_19_	no	0.90	0.47
20	d_20_	no	1.00	0.50	**20**	**d**_**20**_	**yes**	**1.00**	**0.50**

A related approach that has been used more frequently in recent times is **mean average precision **(MAP), where the precision is measured at every point at which a relevant document is obtained and then averaged over all relevant documents to obtain the average precision for a given query. For a set of queries, the mean of the average precision for all queries is the MAP of that information retrieval system.

The Text REtrieval Conference (**TREC**) [14,15] is a yearly event, organized by the US National Institute for Standards and Technology (NIST) to encourage research in information retrieval from large text applications by providing a large **test collection **(a fixed collection of documents, queries, and relevance judgments), uniform scoring procedures, and a forum for organizations interested in comparing their results. A particular track in the biomedical domain (TREC Genomics Track) [16] was started in 2003. In the most recent year of the track (2005), two tasks were included. One assessed **ad hoc retrieval **from topics captured by real biologists, such as finding papers that describe the role of a particular gene in a given disease. For the Genomics track, TREC has provided a special program called ***trec_eval ***to evaluate the performance of each participant's system [17].

## Conclusion

The ease at which users can carry out searches in PubMed and Google should not lull investigators into thinking that information retrieval is an easily learned art, or a mature science. User studies indicate that students and professionals alike are very ineffective and inefficient at retrieving specific items of information via PubMed or web searches [18,19] and the TREC conference has demonstrated that existing IR systems show levels of performance that are far below optimal [[20,21]; see accompanying articles in this journal]. Research is actively underway to learn how to better represent information within documents, and how to index the information using **ontologies **(an ontology is typically a hierarchical data structure containing all the relevant entities, their properties, their relationships, and rules within a certain domain [22]). Many researchers are currently exploring how to go beyond retrieving documents to finding relevant passages or specific relationships mentioned within documents (**information extraction**) (see For Further Reading, Table 5). A variety of web-based services such as KartOO [23] and Vivisimo [24] currently allow the user to visualize or cluster retrieved documents or websites according to their relevance, importance and relationships to each other. Finally, new mathematical models of information retrieval are being explored [25,26] that have yet to be implemented widely.

**Table 5:** For further reading

A. Shatkay H, Feldman R: **Mining the biomedical literature in the genomic era: an overview**. *J Comput Biol *2003, **10**:821-55.
B. Nadkarni PM: **An introduction to information retrieval: applications in genomics**. *Pharmacogenomics J *2002, **2**:96–102.
C. Hersh WR: *Information retrieval: a health and biomedical perspective*. New York: Springer-Verlag, 2nd edition, 2003.
D. Krallinger M, Valencia A: **Text-mining and information-retrieval services for molecular biology**. *Genome Biol *2005, **6**:224.
E. Jensen LJ, Saric J, Bork P: **Literature mining for the biologist: from information retrieval to biological discovery**. *Nat Rev Genet *2006, **7**:119-29.

One may expect that finding information that is present in public databases should be much easier than making new scientific discoveries, yet this task remains a formidable challenge.

## Competing interests

The author(s) declare that they have no competing interests.

## Authors' contributions

W. Z. wrote the first draft and helped modify subsequent drafts. N. S. rewrote the text to make it more accessible to biomedical investigators, and C. Y. double-checked the adequacy and coverage of the topic.

## References

[B1] McKibbon KA, Haynes RB, Dilks CJ, Ramsden MF, Ryan NC, Baker L, Flemming T, Fitzgerald D (1990). How good are clinical MEDLINE searches? A comparative study of clinical end-user and librarian searches. Comput Biomed Res.

[B2] McKibbon KA, Haynes RB, Johnston ME, Walker CJ (1991). A study to enhance clinical end-user MEDLINE search skills: design and baseline findings. Proceedings of the Annual Symposium on Computer Application in Medical Care.

[B3] Insel TR, Volkow ND, Li TK, Battey JF, Landis SC (2003). Neuroscience networks: data-sharing in an information age. PLoS Biol.

[B4] Entrez PubMed. http://www.pubmed.gov.

[B5] Medical Subject Headings. http://www.nlm.nih.gov/mesh/meshhome.html.

[B6] PubMed Tutorial. http://www.nlm.nih.gov/bsd/pubmed_tutorial/m1001.html.

[B7] Swanson DR An introduction to MEDLINE searching. http://arrowsmith.psych.uic.edu/arrowsmith_uic/tutorial/swanson_medlinesearching_2003.pdf.

[B8] Page L, Brin S, Motwani R, Winograd T (1998). The PageRank Citation Ranking: Bringing Order to the Web. Technical Report.

[B9] GoogleGuide. http://www.googleguide.com/google_works.html.

[B10] askMEDLINE. http://askmedline.nlm.nih.gov/ask/ask.php.

[B11] Salton G, Buckley C (1991). Global text matching for information retrieval. Science.

[B12] Srinivasan P (1996). Retrieval feedback in MEDLINE. J Am Med Inform Assoc.

[B13] Aronson AR, Rindflesch TC (1997). Query expansion using the UMLS Metathesaurus. Proceedings of AMIA Annual Fall Symposium.

[B14] Text REtrieval Conference. http://trec.nist.gov/.

[B15] Voorhees E, Harman D, (eds) (2005). TREC: Experiment and Evaluation in Information Retrieval.

[B16] TREC Genomics Track. http://ir.ohsu.edu/genomics/.

[B17] TREC Evaluation Program. http://trec.nist.gov/trec_eval.

[B18] Hersh WR, Hickam DH (1998). How well do physicians use electronic information retrieval systems? A framework for investigation and systematic review. JAMA.

[B19] Wildemuth BM, Moore ME (1995). End-user search behaviors and their relationship to search effectiveness. Bull Med Libr Assoc.

[B20] Hersh WR, Bhupatiraju RT, Ross L, Roberts P, Cohen AM, Kraemer DF Enhancing Access to the Bibliome: The TREC 2004 Genomics Track. J Biomed Discovery Collaboration.

[B21] Cohen AM, Hersh WR The TREC 2004 Genomics Track Categorization Task. J Biomed Discovery Collaboration.

[B22] Gruber TR (1993). A translation approach to portable ontologies. Knowledge Acquisition.

[B23] KartOO visual meta search engine. http://www.kartoo.com/.

[B24] Vivisimo's clustering. http://www.vivisimo.com/.

[B25] Ponte JM, Croft WB (1998). A Language Modeling Approach to Information Retrieval. 21st ACM Conference on Research and Development in Information Retrieval (SIGIR).

[B26] Torvik VI, Weeber M, Swanson DR, Smalheiser NR (2005). A probabilistic similiarity metric for Medline records: a model for author name disambiguation. J Am Soc Information Sci Technol.

[B27] Porter MF (1980). An algorithm for suffix stripping. Program.

[B28] Hersh WR (2003). Information retrieval: a health and biomedical perspective.

[B29] Robertson S, Walker S (1994). Some simple effective approximations to the 2-Poisson model for probabilistic weighted retrieval. Proceedings of the 17th ACM Conference on Research and Development in Information Retrieval (SIGIR), Dublin, Ireland.

[B30] Singhal A, Buckley C, Mitra M (1996). Pivoted Document Length Normalization. Proceedings of the 19th ACM Conference on Research and Development in Information Retrieval (SIGIR).

